# The segment IV approach: a useful method for achieving the critical view of safety during laparoscopic cholecystectomy in patients with anomalous bile duct

**DOI:** 10.1186/s12893-020-00873-x

**Published:** 2020-09-23

**Authors:** Shuichi Fujioka, Keigo Nakashima, Hiroaki Kitamura, Yuki Takano, Takeyuki Misawa, Yu Kumagai, Taigo Hata, Tadashi Akiba, Toru Ikegami, Katsuhiko Yanaga

**Affiliations:** 1 Department of Surgery, The Jikei University Kashiwa Hospital, 163-1 Kashiwa-shita, Kashiwa city, Chiba, 277-0004 Japan; 2 Department of Surgery, The Jikei University School of Medicine, 3-25-8, Nishi-shinbashi, Tokyo, 105-8461 Japan

**Keywords:** Anomalous bile duct, Laparoscopic cholecystectomy, Critical view of safety, Vasculo-biliary injury, Segment IV of the liver

## Abstract

**Background:**

The critical view of safety (CVS) method can be achieved by avoiding vasculo-biliary injury resulting from misidentification during laparoscopic cholecystectomy (LC). Although achieving the CVS has become popular worldwide, there is no established standardized technique to achieve the CVS in patients with an anomalous bile duct (ABD). We recently reported our original approach for securing the CVS using a new landmark, the diagonal line of the segment IV of the liver (D-line). The D-line is an imaginary line that lies on the right border of the hilar plate. The cystic structure can be securely isolated along the D-line without any misidentification, regardless of the existence of an ABD. We named this approach the segment IV approach in LC.

**Methods:**

In this study, we adopted the segment IV approach in patients with an ABD.

**Results:**

From October 2015 to June 2020, 209 patients underwent LC using the segment IV approach. Among them, three (1.4%) were preoperatively diagnosed with an ABD. The branching point of the cystic duct was the posterior sectional duct, anterior sectional duct, or left hepatic duct in each patient. The CVS was achieved in all cases without any complications.

**Conclusion:**

It is a promising technique, especially even for patients with an ABD during LC.

## Introduction

Misidentification is the major course of vasculo-biliary injury (VBI) during laparoscopic cholecystectomy (LC). The common bile duct is frequently mistaken for the cystic duct, while less frequently, an aberrant hepatic duct can be misidentified as the cystic duct [1, 2]. Exposing the proximal one-third of the gallbladder bed and skeletonizing the gallbladder neck before dividing cystic structures are useful processes for avoiding VBI, as proposed by Strasberg et al. in 1995 in the concept of critical view of safety (CVS) [3]. Later, the Tokyo guidelines 2018 (TG-18) advocated the safe steps for achieving the CVS, where the proximal part of the gallbladder is first dissected and the cystic structure is then skeletonized to avoid misidentification [4]. Recently, we used the right posterior corner of the quadrate lobe of the liver, corresponding to the inferior surface of the segment IV, as a specific point to start dissection of the gallbladder. The dissection of the gallbladder is securely performed along the diagonal line (D-line) of the quadrate lobe, represented by the imaginary line connecting the left ventral and right posterior corner of the quadrate lobe of the liver [5]. We named this the segment IV approach. In the present study, we evaluated this approach during LC in patients with an anomalous bile duct (ABD).

## Patients

From October 2015 to June 2020, 209 LCs were performed for cholecystolithiasis or gallbladder polyps using the segment IV approach. Among them, three patients with cholecystolithiasis were preoperatively diagnosed with an ABD.

## Surgical technique and case presentation

**Fig. 1:**
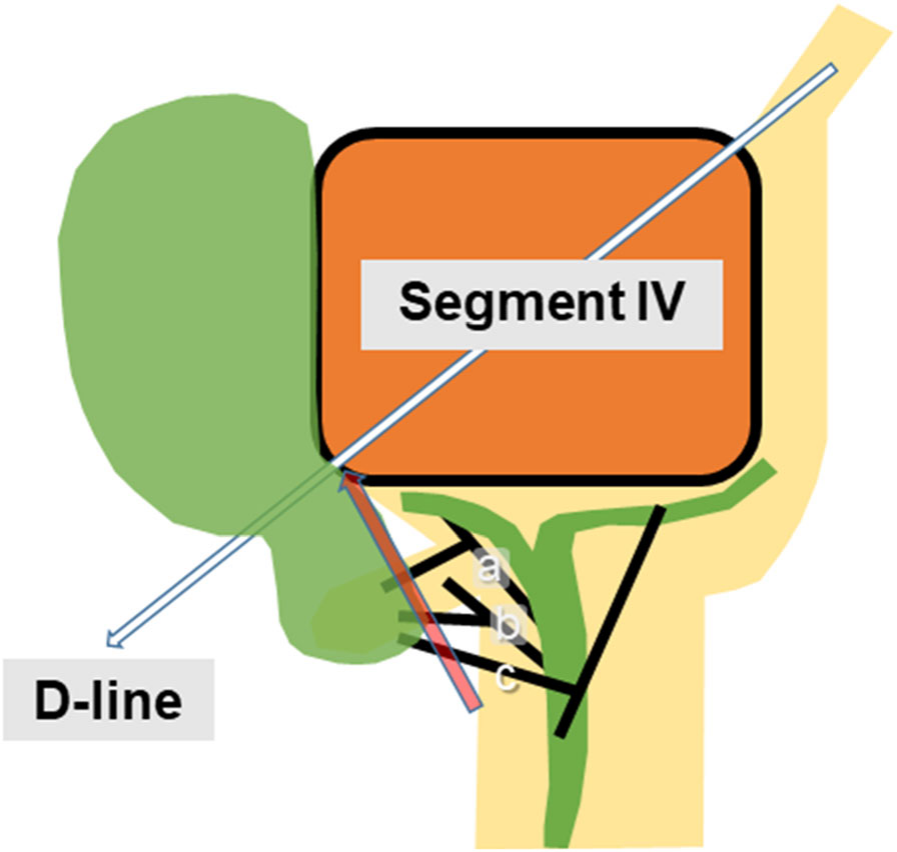
Schematic depiction of the segment IV approach. The gallbladder was first isolated along the diagonal line (D-line) of the segment IV of the liver (white arrow). The cystic structure was then dissected toward the D-line to achieve the critical view of safety (CVS, red arrow). In these case series, anomalous cystic ducts branched off the aberrant anterior hepatic duct (**a**), aberrant posterior hepatic duct (**b**), and aberrant left hepatic duct (**c**), respectively

The concept of the segment IV approach has been published elsewhere [5]. The anatomical variation of the biliary system in relation to the segment IV approach is illustrated in Fig. 1. The gallbladder is first isolated along the imaginary D-line. An operating gauze, which was packed behind the gallbladder along the D-line, acted as the visible endpoint while the cystic structure was dissected to achieve CVS (Fig. 1). Theoretically, the biliary components, including the ABD, are not injured because the dissection of the cystic structure proceeds along the right border of the hepato-duodenal ligament.

**Fig. 2:**
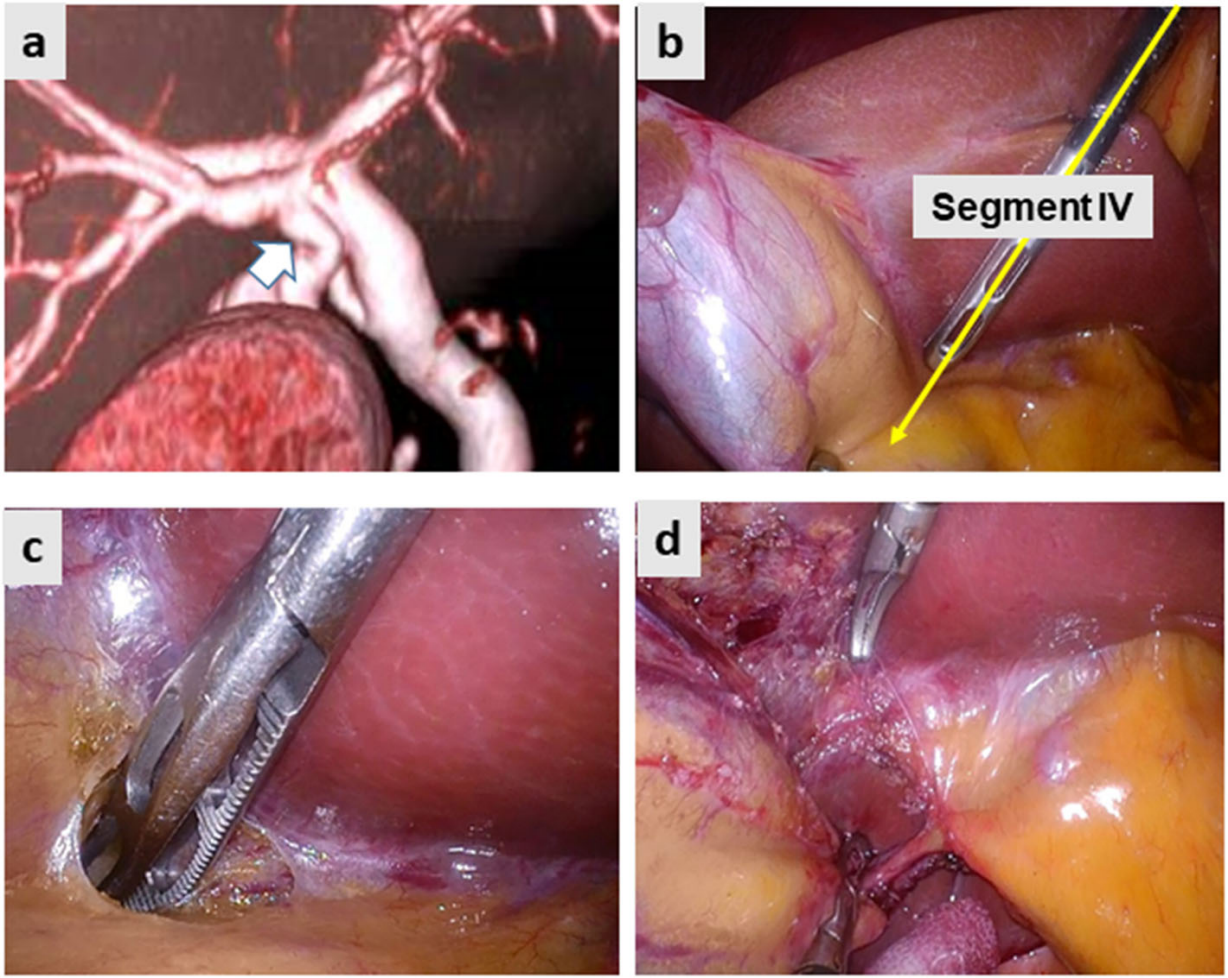
Case 1. The cystic duct branched off the aberrant anterior hepatic duct. **a** Preoperative drip-infusion cholangiography CT. The white arrow indicates the cystic duct branched off the aberrant right hepatic duct. **b** The imaginary diagonal line (D-line) of the segment IV of the liver (yellow arrow) was first identified. **c** The serosa of the gallbladder was incised, and the subserosal layer of the gallbladder wall was dissected along the D-line. **d** Subsequently, the critical view of safety was achieved

**Fig. 3:**
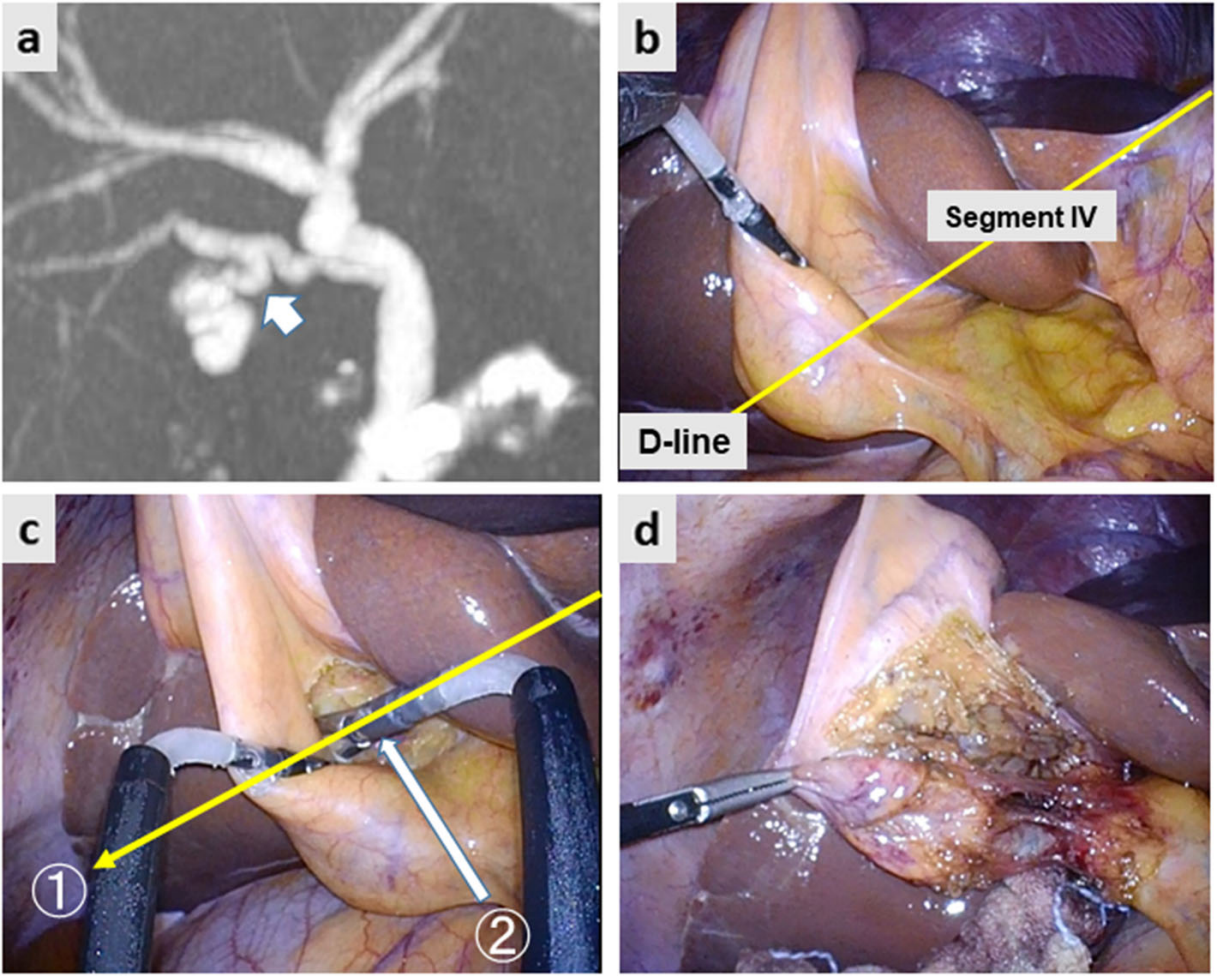
**a** Preoperative drip infusion cholangiography CT image. The white arrow indicates the cystic duct branched off the aberrant posterior sectional duct. **b** The imaginary diagonal line (D-line) of the segment IV of the liver (yellow arrow) was identified. **c**. Dissection of the gallbladder proceeded along the D-line (yellow arrow). The cystic structure was dissected towards the D-line (white arrow). **d** The critical view of safety was achieved

**Fig. 4:**
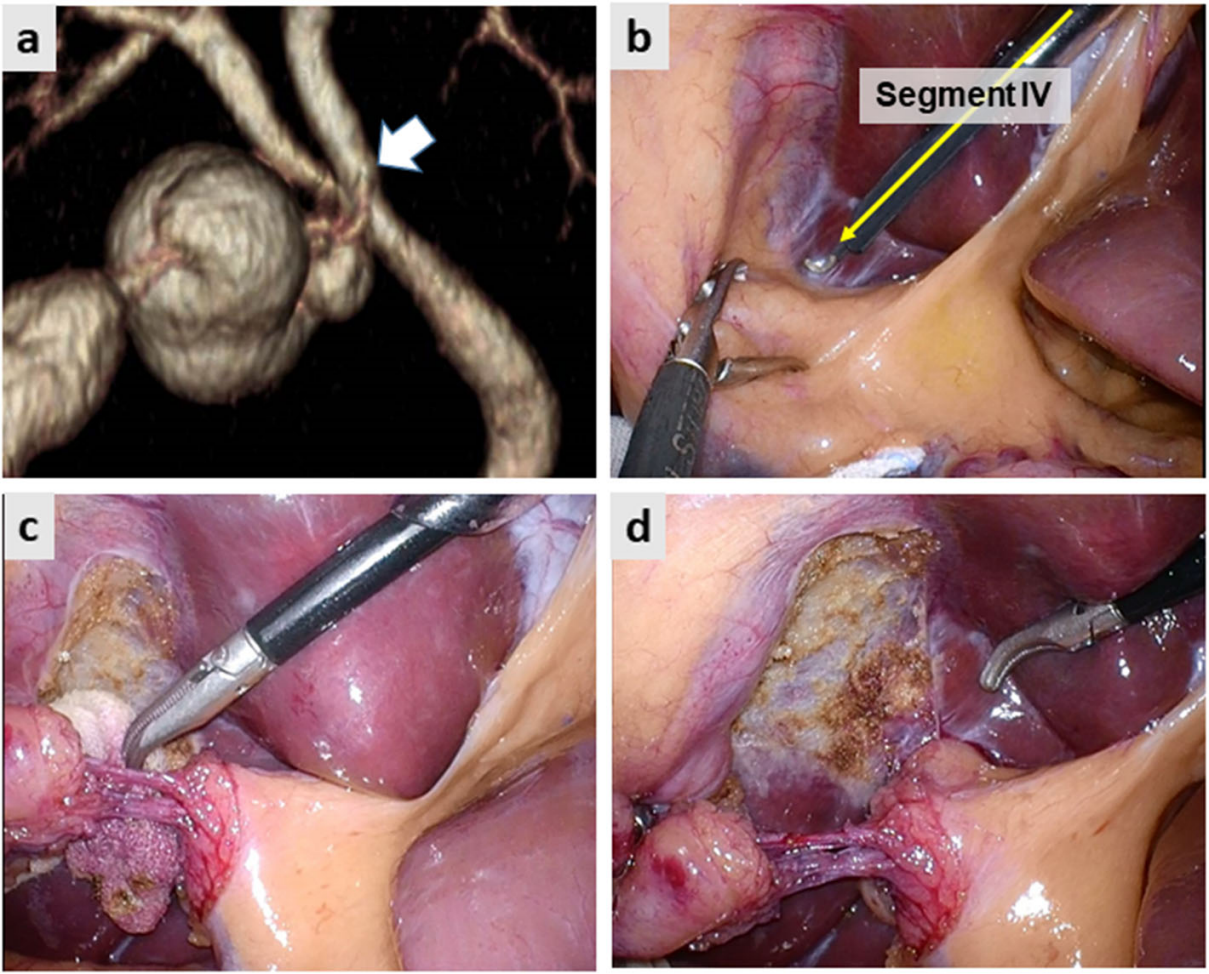
**a** Preoperative drip infusion cholangiography CT image. The white arrow indicates the cystic duct branching off the left hepatic duct. **b** The imaginary diagonal line (D-line) of the segment IV of the liver (yellow arrow) was identified. **c** The dissection of the cystic structure proceeded along the D-line. **d** The critical view of safety was achieved

Fig. 2 demonstrates the preoperative image and intraoperative still pictures of Case 1. Preoperative drip-infusion cholangiography (DIC)-CT indicated that the cystic duct was branched from the anomalous anterior sectional bile duct (Fig. 2a). The two-port LC method was used for this operation. The gallbladder was first isolated along the imaginary D-line (Fig. 2b yellow arrow and Fig. 2c). The cystic structure was skeletonized on the front side of the surgical gauze packed behind the D-line. Subsequently, the CVS was achieved successfully after removing the insolating gauze (Fig. 2d). Figure 3 demonstrates the preoperative image and intraoperative still pictures of Case 2. The preoperative DIC-CT indicated cystic duct branching from the anomalous posterior sectional bile duct, which joined the common hepatic duct (Fig. 3a, white arrow). The single-port LC method was used for this operation. The gallbladder was dissected using angulated dissection forceps along the D-line (Fig. 3b, d) through a multi-access port, which was placed through the umbilicus. The CVS was achieved successfully without exposing the anomalous posterior sectional duct (Fig. 3a). Figure 4a indicates the preoperative DIC-CT image in Case 3, where the cystic duct was branched from the left hepatic duct. The two-port LC method was used for this operation. The gallbladder was isolated and dissected along the imaginary D-line (Fig. 4b and c), and the CVS was achieved (Fig. 4d). No peri- or post-operative complications were encountered. The intraoperative blood loss was minimal, and the operation times in Case 1, 2, and 3 were 82, 75, and 65 min, respectively. All patients were discharged on POD3 without any complications.

## Discussion

As LC became more common, the frequency of VBI was expected to decrease; however, the incidence of VBI remained steady at 0.5% [6, 7]. One explanation for this discrepancy could be the existence of anatomical variations such as the anomalous bile duct. It is universally accepted that in all biliary systems, including the anomalous bile duct converging to the hilar plate system [5, 8–10], the risk of VBI can be decreased by avoiding dissection through the hilar plate. The concept of the CVS have been proposed to avoid misidentification of major vasculo-biliary components during LC [3]. However, standardized landmark, approach or procedure for achieving CVS had not been advocated. Recently, Tokyo Guidelines 2018 described that LC should be performed above the imaged line between the base of segment IV of the liver and the roof of the Rouviére’s sulcus for safe LC [4]. Similar referenced line proposed by Gupta et al. in 2019, in which LC must be done ventral and cephalad to the line joining the roof of the Rouviére’s sulcus and base of segment 4 [11]. We proposed the segment IV approach in 2019, which is based on the operative management for safe LC by dissecting the gallbladder first along the D-line, the right edge of the hilar plate system. D-line lies right side as compared with former two referenced line and the surgical gauze, which is placed along the D-line, plays a useful landmark for safe dissection of hepato-cystic triangle.

As have been described in our original report, the anterior Glissonean pedicle across on the back side of the D-line which is easily identified by using flexible laparoscope, and is secured by using of blunt-tip dissecting forceps. Also D-line lies apart more than 3 mm from the roof of the Rouviére’s sulcus regardless of the shape of caudal surface of the segment IV [5].

The operating gauze, which is isolated along the D-line, acts as a constantly visible landmark to safe dissection. Based on our experience, the D-line corresponds to the narrow segment of the gallbladder neck, which facilitates the isolation of the gallbladder [5]. While the D-line corresponds to proximal root of the cystic plate, bile ducts are not exist on the side of the D-line. Thus, the segment IV approach may be useful for avoiding VBI while performing LCs in patients with an ABD. In this study, the CVS was achieved in all cases without exposing the ABD. Intraoperative still pictures of the CVS indicated that the dissection proceeded to the right border of the hilar plate. The advantage of the segment IV approach is its simplicity. This approach only requires two steps to achieve the CVS: isolation of the gallbladder along the D-line and dissection of the cystic structure towards the D-line. The question will be the advantage of the D-line method as compared to conventional fundus-down cholecystectomy. Strasberg et al. reported that most VBI, especially in inflamed gallbladder, had been caused by fundus-down approach, due to the thickening and shrinking of the cystic plate [12]. We consider the risk of fundus-down approach that lack of the landmark to which the gallbladder is to dissect. As long as the D-line is secured by surgical gauze, hepato-cystic triangle is dissected safely without misidentification. Once the gallbladder is isolated by surgical gauze, the CVS can be achieved by removing fat and fibrous tissue in front of the gauze. Thus, the segment IV approach enables a surgeon to achieve the CVS simply, regardless of the presence or absence of an ABD.

The segment IV approach has some limitations. The approach is not applicable in cases where the margin of the gallbladder cannot be recognized for anatomical identification of the D-line due to inflammatory adhesion with surrounding structures [5]. Although we have not experienced such complications in the current study, which is better to convert to open surgery, since laparoscopic dissection of the gallbladder from the lateral side can lead to injury of vasculo-biliary components.

In conclusion, it is a promising technique, even in patients with an ABD.

## Data Availability

Not applicable.

## Abbreviations

CVS: critical view of safety
LC: laparoscopic cholecystectomy
ABD: anomalous bile duct
D-line: diagonal line of the segment IV of the liver
VBI: vasculo-biliary injury
TG-18: Tokyo guidelines 2018
DIC: drip-infusion cholangiography
